# Supplementary material for: An Optimized Checkerboard Method for Phage-Antibiotic Synergy Detection
Source: Viruses. 2022 Jul 14;14(7):1542. doi: 10.3390/v14071542 (PMC9319746; doi:10.3390/v14071542)
Supplement: Supplementary file 1 [file viruses-14-01542-s001.zip › viruses-1806801-supplementary.pdf]

Table S1. MIC of antibiotics.

	MIC (μg mL^{-1})			
Strain	CIP	GEN	CRO	OXA
P. aeruginosa UCBPP-PA14	0.125	0.50	32.00	NA
S. aureus ATCC 43300	0.50	32.00	NA	2.00

NA not applicable.

Table S2. MIM of bacteriophages.

	MIM		
Strain	JG024	MSA6	SES43300
P. aeruginosa UCBPP-PA14	1	NA	NA
S. aureus ATCC 43300	NA	0.001	0.0001

NA not applicable.
